# CBCT and Micro-CT analysis of the mandibular first premolars with C-shaped canal system in a Chinese population author

**DOI:** 10.1186/s12903-023-03271-w

**Published:** 2023-09-30

**Authors:** Yimeng Zhang, Xunben Weng, Yu Fu, Xuekai Qi, Yihuai Pan, Yu Zhao

**Affiliations:** 1 School and Hospital of Stomatology, Wenzhou Medical University, Wenzhou, Zhejiang Province People’s Republic of China; 2 Department of Endodontics, School and Hospital of Stomatology, Wenzhou Medical University, Wenzhou, Zhejiang Province People’s Republic of China

**Keywords:** C-shaped canal, Mandibular first premolar, Micro-CT, CBCT, Morphology

## Abstract

**Objectives:**

The purpose of this study was to survey the prevalence of C-shaped root canal system in mandibular first premolar in Chinese population by reading Cone-beam computed tomography (CBCT) images and to analyze its anatomical characteristics by CBCT and Micro–computed tomography (Micro-CT).

**Methods and materials:**

The prevalence and the morphologic features of C-shaped root canal system were evaluated by observing CBCT images of 760 patients (1520 mandibular first premolars). 66 mandibular first premolars with C-shaped root canal system were scanned by Micro-CT. The morphologic features including radicular groove, C-shaped root canal categories in the cross-sections and in the 3D models, accessory and connecting canals, apical foramina and accessory foramina, were analyzed using image software.

**Results:**

C-shaped root canal system was identified in 16.9% of mandibular first premolars. The minimum mesial wall thickness most commonly occurred at the lingual site (69.7%). Regarding to the cross-sectional classification, the maximum was C2 (41.5%). In the 3D classification, the most common was S (34.8%). Accessory canals were observed in 36.4% of the samples and occurred mostly in the middle and apical regions. 42.4% samples had 1–3 variable connecting canals, and 40.9% samples had only one apical foramen.

**Conclusions:**

The incidence of C-shaped root canal system in mandibular first premolars was 16.9% in the Chinese population. The anatomy was very complex and variable, mostly distributed in the middle and apical regions of the root canal. The mesial wall of C-shaped canal was extremely thin on the lingual side.

## Introduction

The knowledge about the anatomy of root canal system is the foundation of proper debridement and three-dimensional obturation, which is closely related to the success of the endodontic therapy.

Mandibular first premolar architecture varies, with a considerable proportion of C-shaped canal configurations [1, 2]. The main anatomic feature of C-shaped canal system is the presence of fins or webs connecting individual canals. The C-shaped canal system of mandibular first premolars is typically composed of two canals, which combine partially or completely to form a C-shaped strip in horizontal cross section. The prevalence of the characteristic C-shaped canals in the mandibular first premolars have been reported to be 12.5-67.47% in Asia and South America, which is higher than other areas [3, 4].

Because of its noninvasion, cone-beam computed tomography (CBCT) has been widely employed to examine the complex root canal anatomy in clinical practice. The usual axial slices of CBCT, 108–300 μm [2, 3, 5–10], are too large to give exact images of the apical root canal. Although the micro-computed tomography (Micro-CT) can only utilize in vitro, the method is capable to offer accurate information about the canal anatomy in details. Therefore, Micro-CT is also regarded as the gold standard in the root canal anatomical studies [11].

The purpose of this study was to investigate the prevalence of mandibular first premolar with C-shaped canal in Chinese population and to analyze its anatomic characteristics using CBCT and Micro-CT scanning.

## Method and materials

### Sample selection

CBCT images of mandibular first premolars were collected from patients who had undergone scanning for diagnostic purposes between January 2020 and December 2022. CBCT scans were mainly obtained for implant surgery, surgical removal of impacted teeth or orthodontic treatment. Therefore, the subjects in the study were not exposed to unnecessary radiation. The image data were all from a native Chinese population. The inclusion criteria of the mandibular first premolar was as follows: the apex fully developed without any caries, fracture, resorption and calcification, and no accepted endodontic treatment. Vague or blurred CBCT images were excluded. Finally, 1520 mandibular first premolars were selected as the samples into this study. The protocol in this study were approved by the Ethics Committee of the School of Stomatology of Wenzhou Medical University (WYKQ2021007).

The extracted mandibular first premolars were collected from a native Chinese population. The reasons of extraction were unrelated to this study. Including criteria were the same as above-mentioned. The teeth were clearly identified as mandibular first premolar saccording to the morphologic criteria [12]. The samples were ultrasonically cleaned to remove attached soft tissues and calculus and then stored in 10% neutral buffered formalin. All samples were scanned by CBCT (Newtom VGi. Cefla, Imola, Italy)to identify the C-shaped canal anatomy. Finally, 66 mandibular first premolars were selected to and scanned using Micro-CT.

In this study, the C-shaped root canals were identified and categorized based on Melton’s classification with the modifications proposed by Fan et al. [13]. The canals were classified as follows: continuous C-shaped root canals (C1), interrupted C-shaped root canals resembling a semicolon (C2), and two separated root canals with round, oval, or flattened morphology (C3). When the canal configuration showed as the aforementioned morphology in at least one cross-section, the sample would be identified as a mandibular first premolars with C-shaped root canal.

### CBCT image analysis

The data were captured using Newtom VGi (Cefla, Imola, Italy)set at 110 kV, 3mA, 150 × 150 mm field of view, and 250 µm^3^ voxel size. The images were observed through NNT Viewer (12.1.0.0, Cefla, Imola, Italy). A postgraduate specialized in endodontics evaluated the CBCT images in the study. To assess the intra-observer reliability, 100 selected CBCT images of mandibular first premolars with diverse root canal morphology were examined twice with a one-month interval between assessments. The kappa statistic value of 0.907 signified a high degree of agreement.

The patient’s age and gender from CBCT image file were recorded. The prevalence of C-shaped canal in different gender and age groups was calculated and compared. The presence or absence of root surface groove, as well as its distributing direction (buccal, lingual, mesial and distal) in the root surface was recorded. A brief examination of the crosssection was executed to examine C-shaped canals, in the unilateral and bilateral occurrence. A thorough examination was conducted to identify the cross-sectional classification and the specific zone where the C-shaped canals occurred, such as the coronal, middle, or apical zones of the roots.

### Micro-CT image analysis

The 66 selected samples were scanned by Micro-CT (SkyScan 1176; Bruker-micro-CT, Kontich, Belgium). The scanning parameters used were 90 kV, 270 µA, 0.1 mm Cu filter, 17.54 µm^3^ pixel size, and 360° rotation with a rotation step of 0.5°. The raw data were reconstructed by NRecon (v 1.6.10.4; Bruker-micro-CT, Kontich, Belgium) and then exported in TIFF format files.

The images were segmented by DataViewer (v 1.5.6.2; Bruker-micro-CT, Kontich, Belgium). Qualitative and quantitative analyses as followed were preformed and 3D models were created in STL format using the CTAn software (v 1.20.3.0; Bruker-micro-CT, Kontich, Belgium). The canals and roots were observed by using the CTVol (v 2.3.2.0; Bruker-micro-CT, Kontich, Belgium) software and the CTVox software (v 3.3.0.0; Bruker-micro-CT, Kontich, Belgium), respectively.

**Fig. 1:**
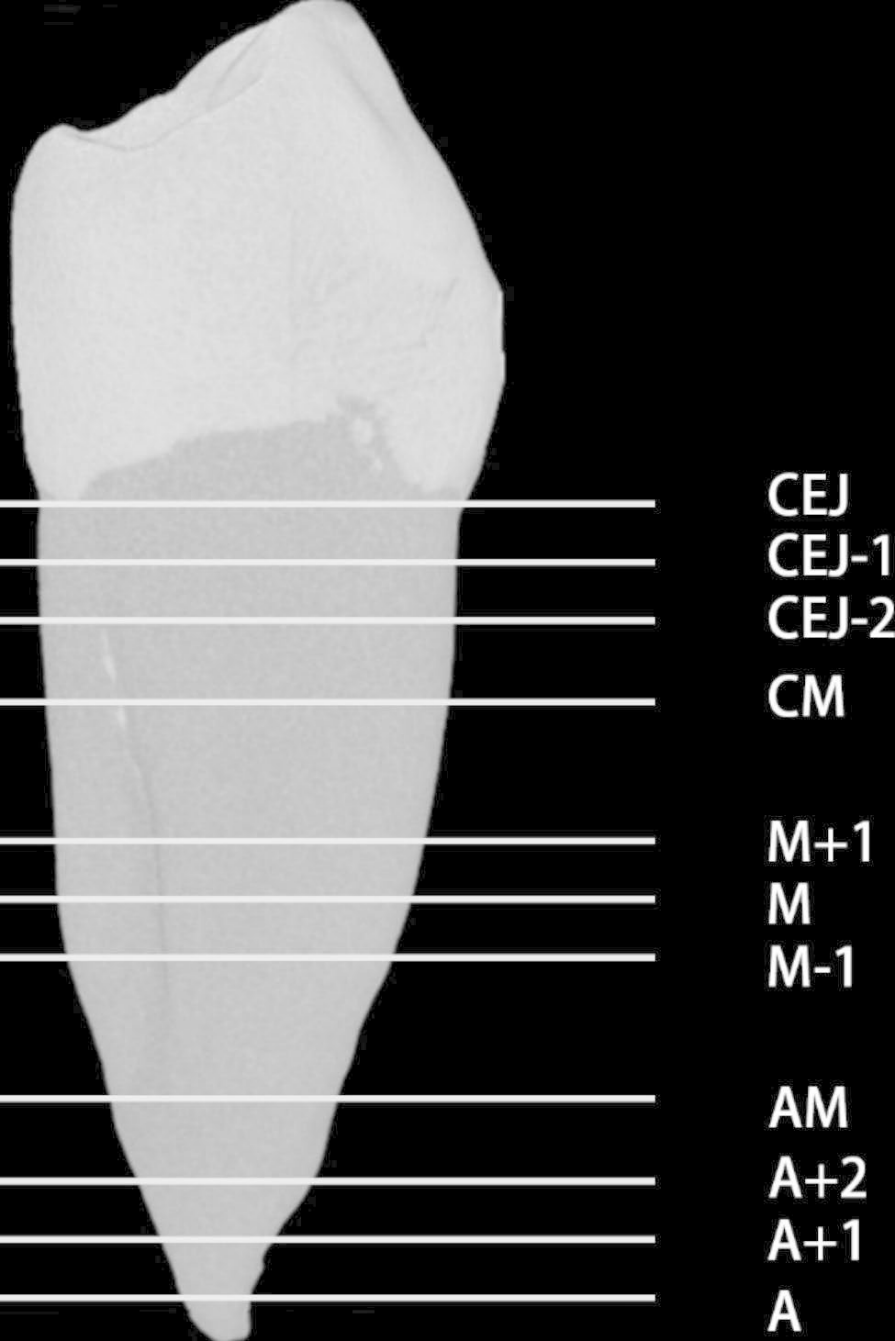
Measurement levels over the length of the root in the Micro-CT images CEJ, the cementoenamel junction; CEJ-1, 1 mm below the cementoenamel junction; CEJ-2, 2 mm below the cementoenamel junction; CM, the junction of the coronal third and middle third of the root; M + 1, 1 mm above the middle of the root; M, the middle of the root; M-1, 1 mm below the middle of the root; AM, the junction of apical third and middle third of root; A + 2, 2 mm above the apical; A + 1, 1 mm above the apical; and A, the apical

**Fig. 2:**
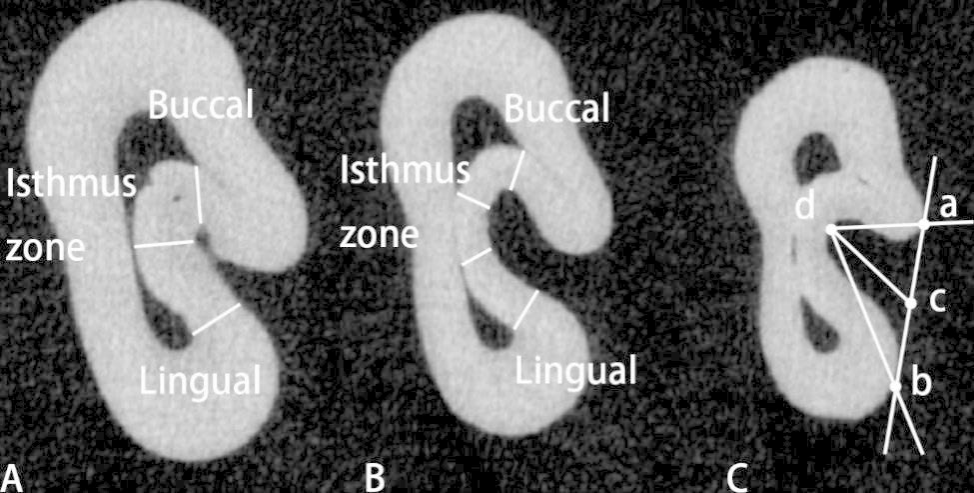
Measurement of the minimum mesial wall thickness and the depth and angle of the radicular groove in C-shape canal cross-section (**A**) Minimum mesial wall thickness measurement site in C1. (**B**) Minimum mesial wall thickness measurement site in C2, C3. (**C**) Line ab is the tangent line of the outer edge of the radicular groove; point c is the midpoint of the tangent points (a and b); the distance from point c to the bottom of the groove (point d) is the depth; ∠adb is the groove angle

**Fig. 3:**
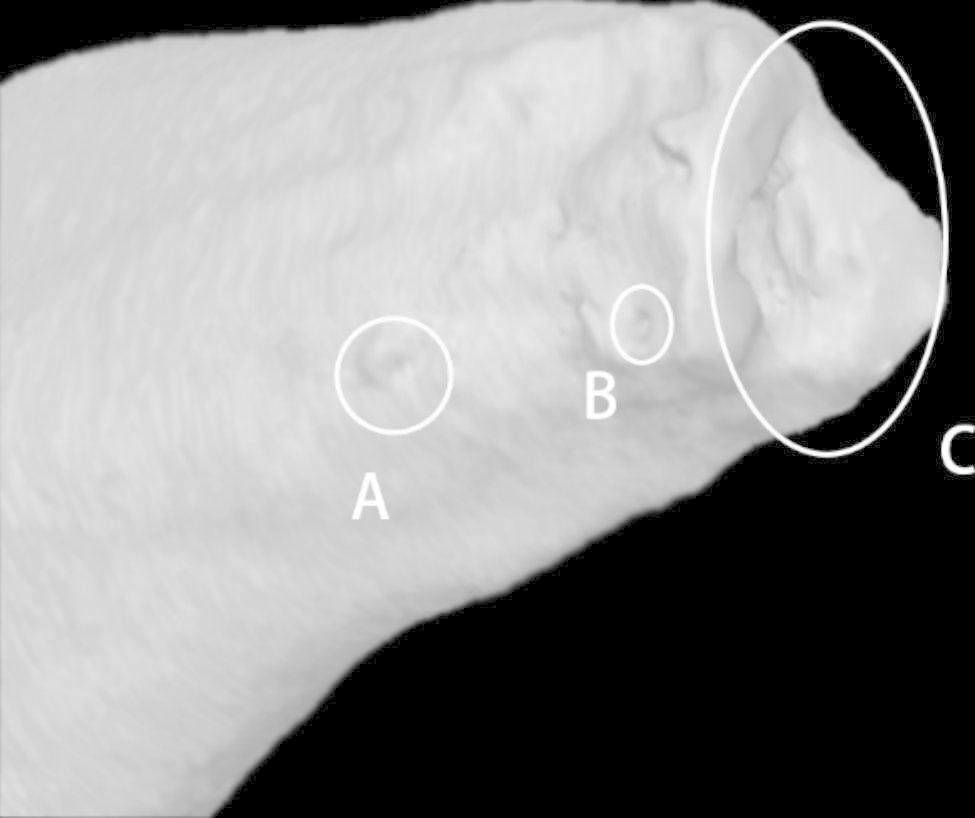
Accessory foramina in the 3D model of a root (**A**) (**B**) accessory foramina. (**C**) apical foramen

1. The radicular groove:
  - The number of radicular grooves was recorded. The depth and angle [14] of the radicular grooves from the cementoenamel junction (CEJ) to the apex [13] (Fig. 1) was measured. And their distributing sites on the root surface were analyzed.
    The cross-sections were selected at the following levels: the cementoenamel junction (CEJ); 1 mm apically to the CEJ (CEJ-1); 2 mm apically to the CEJ (CEJ-2); the junction of the coronal third and middle third of root (CM); 1 mm coronally to the middle of the root (M + 1); the middle of the root (M); 1 mm apically to the middle of the root (M-1); the junction of the apical third and middle third of root (AM); 2 mm apically to the anatomic apex (A + 2); 1 mm apically to the anatomic apex (A + 1); and the anatomic apex (A).
  - The minimum mesial wall thickness of the cross section with C-shaped configuration (Fig. 2) was recorded according to the method of Chai et al. [15].
  The measurement sites were classified as buccal, which included the buccal side of C1 and the buccal canal of C2 and C3; isthmus, which included the constricted region of C1 and the equivalent portion of C2 and C3; and lingual, which included the lingual side of C1 and the lingual canal of C2 and C3.
2. 2D classification of C-shaped canal:
  The 2D classification of C-shaped canal in the cross-sections was recorded from CEJ to A + 1 layer.
3. The 3D morphology of C-shaped canal:
  The 3D classification of C-shaped canal in the mandibular first premolar was proposed by Fan et al. [16]. Based on the continuous variation of root canal morphology in the axial direction, the classification method includes 4 categories: Continuous C-shaped canal only (C); Semilunar buccal canal only (S); Combination of continuous C-shape and semilunar buccal canal (CS); C-shaped canal interrupted by non-C-shaped canal (CS + N).
4. The accessory and connecting canals:
  The number and location of the accessory and connecting canals in the root various regions was recorded.
5. The apical foramina:
  The number of apical foramina of each sample was recorded. The major and minor diameter was measured based on the method described by Wolf et al. [17]. When multiple apical foramina exist, the difference in diameter should not be more than 0.2 mm. Otherwise, the smaller one is considered to be the accessory foramen.
6. The accessory foramina:
  The number and location of the accessory foramina in the apex was recorded (Fig. 3).

### Statistical analysis

Each measurement was repeated three times, and the mean was taken. The statistical analyses were preformed using SPSS (v 23; SPSS Inc., Chicago, IL, USA). The normal distribution of the data was confirmed by the Shapiro–Wilk test. The Kruskal-Wallis test or the Mann-Whitney U test was used to analyze the intergroup significance. Spearman’s multiple linear regression tested inter-parameter dependence. The differences were considered to be significant if the *p* value was less than 0.05.

## Results

### CBCT findings

In the 1520 mandibular first premolar images, 29.41% (447/1520) had radicular groove and 16.9% (257/1520) had the C-shaped canal. The examinations were collected from 760 patients (343 males and 417 females) with an average age of 32.2 years old.

The C-shaped root canal prevalence in relation to gender and age was shown in Table 1. 119 of the 257 mandibular first premolars with C-shaped canal were from males and 138 from females. The difference of C-shaped canal prevalence between gender was statistically nonsignificant (*p*>0.05). In various age groups, the youngest (15y-20y, 25.5%) group showed significantly higher prevalence than any other groups. And the younger (21y-40y, 16.6%) group also showed significantly higher prevalence than the oldest (>60, 7.6%) group. Regarding the unilateral and bilateral occurrence, 12.9% (98 of 760 subjects) had bilateral C-shaped canals in the population. If calculated using the individuals with C-shaped canals as the common denominator, the prevalence of bilateral C-shaped canals increased to 61.6% (98 of 159 subjects). This concurrent appearance of C-shaped canals was statistically significant (*p*>0.05).

**Table 1:** C-shaped root canal prevalence in CBCT images in relation to gender and age (n, %)

C-shaped	Present	Absent
**Sex**		
Male	119(17.3)^A^	567(82.7)
Female	138(16.5) ^A^	696(83.5)
**Age**		
15y-20y	71(25.5) ^A^	207(74.5)
21y ~ 40y	114(16.6) ^B^	574(83.4)
41y ~ 60y	65(14.1) ^BC^	397(85.9)
>60y	7(7.6) ^C^	85(92.4)
**Total**	**257(16.9)**	**1263(83.1)**

Columns: for comparisons among the different gender and age, different uppercase superscripts in a single column represent significant differences in prevalence between genders and among the age groups (*p* < 0.05)

**Fig. 4:**
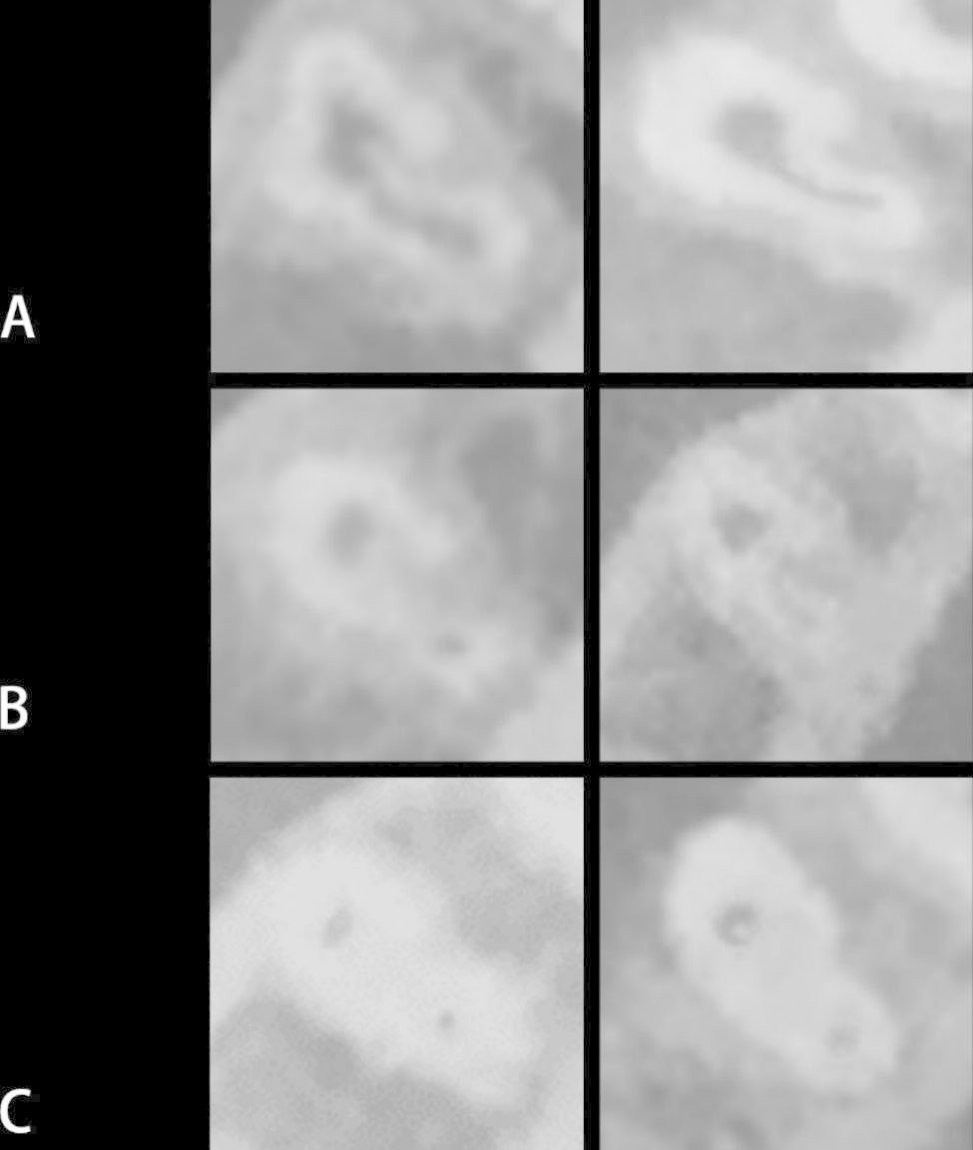
Representative CBCT images of C-shaped root canal in mandibular first premolars (**A**) C1; (**B**) C2; (**C**) C3

The cross-sectional types of C-shape canals in the various root regions were showed in Table 2; Fig. 4. The most common type in the cross-section of C-shaped canal was C2 type (51.5%). The proportion of C-shaped canals located in the apical region (59.7%), is the highest (*p* < 0.05). The C-shaped configuration was rarely present in the coronal region (1.8%). The most common type was C2 in the middle region, and in the apical region (79.5%).

**Table 2:** C-shaped root canal cross-sectional types at the various root regions (n, %)

Cross-sectional types	Axial region			Total
	Coronal	Middle	Apical	
C1	-	20(52.6) ^Aa^	18(47.4) ^Ab^	38(9.7)^A^
C2	7(3.5) ^a^	99(49.3) ^Bb^	95(47.3) ^Bc^	201(51.5)^B^
C3	-	31 (20.5) ^Ca^	120(79.5) ^Cb^	151(38.7)^C^
**Total**	7(1.8)^a^	150(38.5)^b^	233(59.7)^c^	

Rows: for comparisons among the different root canal regions, different lowercase superscripts in a single row represent significance differences among the different root canal regions (*p* < 0.05)

Columns: for comparisons among the different C-shaped canal types, different uppercase superscripts in a single column represent significant differences among the different C-shaped canal types (*p* < 0.05)

### Micro-CT findings

1. The radicular groove:

The number of radicular grooves in a sample varied from 1 to 5, and 37.9% (25/66) of samples had 3 radicular grooves. The radicular grooves were most common on the mesial surface of the root (61.4%).

The distribution of the measurement sites of minimum mesial wall thickness was displayed in Table 3.

**Table 3:** Measurement sites of the minimum mesial wall thickness in Micro-CT cross-sectional images (n, %)

Location	Buccal	Isthmus zone	Lingual	Total
CM	-	-	5(100.0)	5(2.7)
M + 1	3(8.3)	7(19.4)	26(72.2)	36(19.1)
M	7(15.6)	7(15.6)	31(68.9)	45(23.9)
M-1	7(13.5)	9(17.3)	36(69.2)	52(27.7)
AM	5(13.9)	7(19.4)	24(66.7)	36(19.1)
A + 2	3(27.3)	2(18.2)	6(54.5)	11(5.9)
A + 1	-	-	3(100.0)	3(1.6)
Total	25(13.3)^a^	32(17.0)^b^	131(69.7)^b^	188(100.0)

Rows: for comparisons among the different measurement sites of the minimum mesial wall thickness, different lowercase superscripts in a single row represent significance differences among the different measurement sites (*p* < 0.05)

A total of 188 cross-sections with C-shaped canal were identified from CM to A + 1 layer of 66 samples. The highest proportion of C-shaped canals appeared in M-1 layers, reaching 27.7% and the lowest in A + 1 1.6%. The minimum mesial wall thickness frequently presented in lingual site 69.7% (131/188) and rarely in buccal site 13.3% (25/188) (*p* < 0.05).

The minimum mesial wall thickness, the depth and angle of radicular groove was shown in Table 4. The depth and angle median of the radicular groove reached the extreme value in M-1 layer.

**Table 4:** Minimum mesial wall thickness, depth and angle of radicular grooves

Location	Mesial wall thickness (mm)	Depth of groove (mm)	Angle of groove (°)
CM	0.87(0.67–1.06)	0.84(0.48-1.00)	120.55(104.31–128.10)
M + 1	0.78(0.70–0.86)	1.04(0.79–1.37)	101.03(82.77- 122.84)
M	0.68(0.61–0.75)	1.29(0.99–1.67)	93.61(72.55–98.30)
M-1	0.63(0.59–0.71)	1.32(1.01–1.64)	82.80(72.60–95.90)
AM	0.61(0.55–0.66)	1.21(0.91–1.50)	89.50(70.09–98.43)
A + 2	0.48(0.31–0.53)	0.97(0.64–1.24)	87.62(62.69-129.68)
A + 1	0.37(0.29–0.41)	0.53(0.49–0.73)	110.02(102.49-147.62)

The correlation analysis on the mesial wall thickness as the dependent variable was presented in Table 5. The cross-section level (*p* < 0.05, *r*=-0.433), location (*p* < 0.05, *r* = 0.396), depth (*p* < 0.05, *r*=-0.371) and angle (*p* < 0.05, *r* = 0.438) of radicular groove was related to the mesial wall thickness.

**Table 5:** Multiple non-linear regression analysis of mesial wall thickness and different factors

Independent variables	Mesial wall thickness	
	*r*	*p value*
Cross section level	-0.433	< 0.05
C-shaped canal configuration	0.013	0.862
Location of the measurement	0.396	< 0.05
Mesial wall thickness	1.000	-
Depth of groove	-0.371	< 0.05
Angle of groove	0.438	< 0.05

2. The 2D classification of C-shaped canal:

In the cross-sectional images of Micro-CT, the proportions of C1, C2, and C3 are 35.1%, 41.5%, and 23.4%, respectively. This result was consistent with the CBCT analysis.

**Fig. 5:**
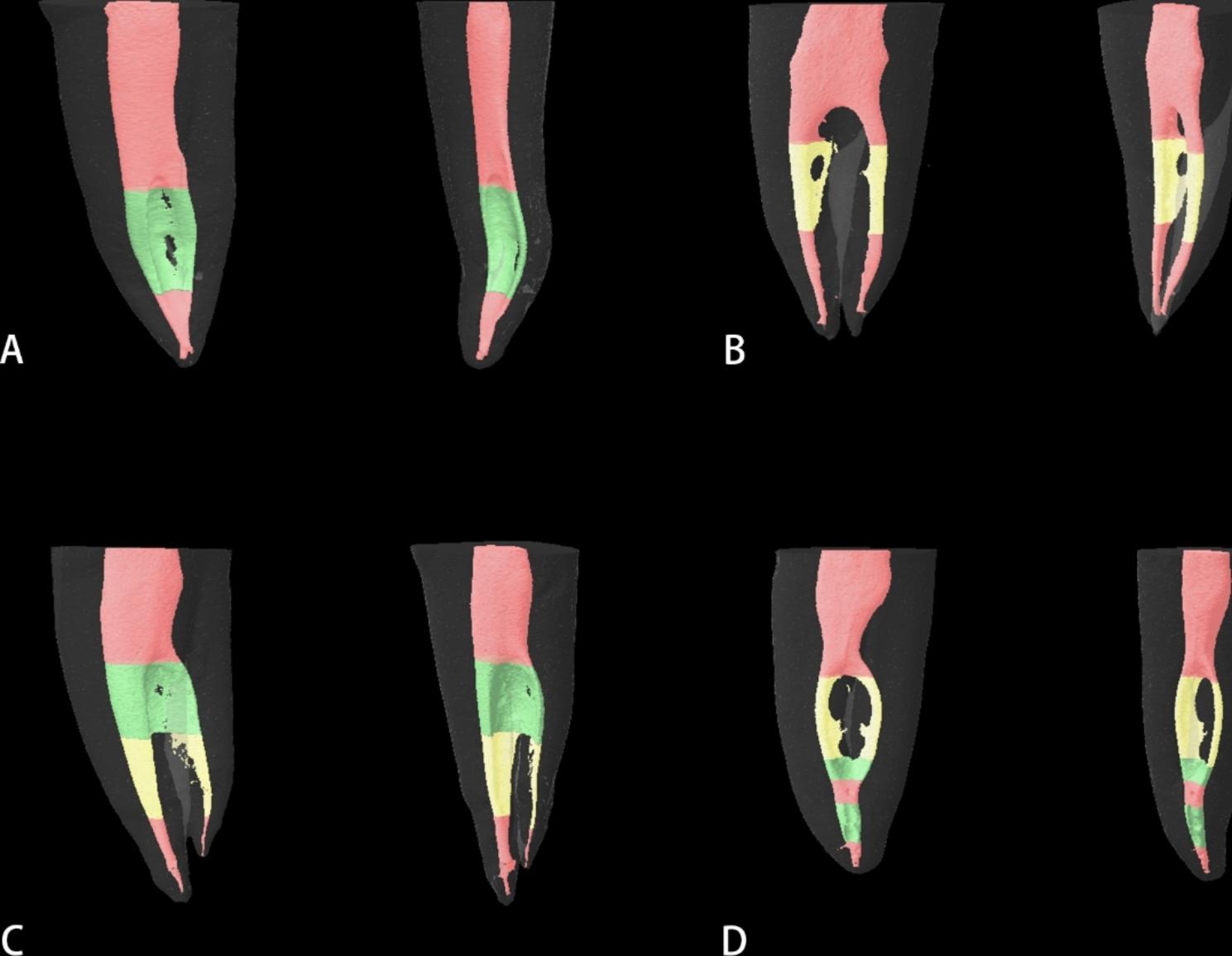
Three-dimensional C-shaped root canal classification in proximal and 45° angle views Red: non-C-shaped canal; Green: continuous C-shaped canal; Yellow: semilunar buccal canal. (**A**) Continuous C-shaped canal only;(**B**) Semilunar buccal canal only;(**C**) Combination of continuous C-shape and semilunar buccal canal;(**D**) C-shaped canal interrupted by non-C-shaped canal

3. The 3D morphology of C-shaped canal:

The representative 3D morphology of each classification was shown in Fig. 5. The proportion from high to low was S (34.8%), CS (33.3%), C (24.2%), and CS + N (7.6%), respectively. The difference between CS + N type and other types was statistically significant (*p* < 0.05).

4. The accessory and connecting canals:

The location and number of accessory canals and connecting canals was presented in Table 6. The number of accessory canals and connecting canals differed from 1 to 3 among different samples. The occurrence rate of accessory canals was 36.4% in all the specimens. The accessory canals were mainly located in the middle regions (59.0%) and rarely in the coronal regions (5.1%). Connecting canals were observed in 28(42.4%) specimens. The samples with one connecting canal were the most commonly observed (81.3%). The connecting canals were more frequently located in the apical third (59.4%).

**Table 6:** Location and number of accessory canals and connecting canals (n, %)

Accessory canals	Number of accessory canals				Number of connecting canals		
	1	2	3		1	2	3
Coronal	2(100.0)	-	-		-	-	
Middle	19(82.6)	3(13.0)	1(4.3)		11(84.6)	1(7.7)	1(7.7)
Apical	10(71.4)	4(28.6)	-		15(78.9)	2(10.5)	2(10.5)

5. The apical foramina:

One anatomical foramen was the most frequently observed in specimens 27(40.9%). The larger number of the foramina there was, the smaller the minor (*p* < 0.05, *r*=-0.591) and the major (*p* < 0.05, *r*=-0.534) diameter there was (Table 7).

**Table 7:** major diameter and number of apical foramina

Foramina	1	2	3
**Minor (mm)**	0.25(0.20–0.26)^a^	0.14(0.12–0.16)^b^	0.12(0.09–0.13)^c^
**Major (mm)**	0.34(0.31–0.38)^a^	0.26(0.24–0.30)^b^	0.26(0.25–0.27)^b^
**Number(%)**	27(40.9)	26(39.4)	13(19.7)

Rows: for comparisons among the different apical foramina numbers, different lowercase superscripts in a single row represent significance differences in each diameter among the different apical foramina numbers (*p* < 0.05)

6. The accessory foramina:

Accessory foramina were found in 45(68.2%) specimens (Table 8). The numbers of accessory foramina differed from 1 to 6 in the specimens. The samples with 1 accessory foramen accounted for the highest proportion (25.8%), whilst the samples with 6 foramina had the lowest proportion (1.5%).

**Table 8:** Number of samples with accessory foramina (n, %)

Accessory foramina	Number of samples (n, %)
1	17(25.8)
2	13(19.7)
3	8(12.1)
4	3(4.5)
5	3(4.5)
6	1(1.5)
**Total**	**45(68.2)**

## Discussion

In the present study, the prevalence of C-shaped canal configuration in the mandibular first premolars in a Chinese population was 16.9%, which was similar to the range of 12.5–18% reported by previous studies [11, 18]. It was the region and the race [2, 4, 11, 13, 16, 19, 20] that possibly varied the prevalence in mandibular first premolars as reported.

CBCT images were collected from 760 patients in Chinese population, of whom 343 were males and 417 were females. And the prevalence of C-shaped canals was 17.3% in males and 16.5% in females, respectively. There was no statistically significant difference between genders. This result was consistent with the findings of most previous studies, Martins et al. [19] reviewed the prevalence, odds ratio and heterogeneity of C-shaped root canal system in mandibular first premolar by gender, where no significant differences were observed (*p* > 0.05). In this study, the prevalence of C-shaped root canal system tended to decrease along with the increase of age. The result may be attributed to the fact that the samples from the 15–20 years old group had larger canals and pulp chambers with insufficient developed roots [21, 22], which were more easily observed at the same resolution. Whereas, natural physiological aging on the one hand, and on the other hand, long-term environmental stimulation, such as occlusal trauma and periodontal disease, could modify the deposition of dentine [23] and root canal system morphology [24]. These factors together lead to thickening of the root canal wall and narrowing of the root canal. These canals were more blurred at the same resolution, and the characteristic C-shaped root canal morphology was more difficult to discern.

Amongst 1520 CBCT scanning files, C2 showed the highest proportion (51.5%) and distributed mainly in the middle (49.3%) and the apical (47.3%) regions. C3 configuration accounted for 38.7% and mainly distributed in the apical (79.5%) regions. These results suggested that root canal bifurcations were typically located in the lower and middle and apical segments, which were not visible with the naked eye, and that lingual root canals were more curved and possessed a distinct presence of dentin cusp collars and a narrower diameter than buccal root canals. Similar results were obtained in a study by Ordinola-Zapata et al. [4] They found that C1 and C2 configurations were more prevalent in the coronal and middle of the roots, and C3 were more common at the apical region. The canal anatomy was more prone to complexity towards the apical direction.

In the present study, only 36.4% (8/22) of CS type C-shaped canals appeared the buccal and lingual canals combining together in the 3D models constructed using Micro-CT. And in most of CS type, the buccal and the lingual canals were still present separately, increasing the anatomical complexity of the mandibular first premolar in the middle and apex. According to Li et al. [25], 69% of the Vertucci V class’s mandibular first premolars bifurcated in the middle of the root to form a lingual root canal. In the proximal directions, the lingual root created a modest angle with the buccal root, while an average 33.54° angle existed in the buccolingual direction, with 77% of the samples considerably twisted. Therefore, root canal omissions usually occurred during orifice probing in clinical practice. Some operating skills would be beneficial to the clinicians take CBCT preoperatively to understand the morphology of the canal system comprehensively, pre-curving K files to explore the canal orifice, utilizing the magnification and illumination effects of a dental microscope, and removing the dentine collar with the ultrasonic devices to expose the lingual canal.

The Micro-CT analyses in this study revealed that C-shaped canals were more prevalent from M + 1 to AM layer of the canal, which were inconsistent with the CBCT results. The most likely reason was the huge disparity in accuracy between CBCT and Micro-CT examination. The voxel size of CBCT (250 μm) was larger than the diameter of the middle and apical root canal [26, 27]. According to the Shannon-Nyquist theory, when 1/2 the size of an object was larger than the resolution of CBCT, the image of the object could be observed in CBCT [28]. Moreno et al. [29] reported a narrower diameter of 0.24 ± 0.10 mm at 3 mm from the root apex measured by Micro-CT (voxel size 17 μm), at which time some of the tiny root canals could not be easily distinguished, creating inaccuracies.

Previous studies on mandibular second molars have shown that the wall of the C-shaped root canal was thinner on the sides near the radicular groove than on the remaining side [15, 30, 31]. In the present study, the cross-sections of C-shaped root canal were analyzed. Usually, the lingual mesial wall was the thinnest (69.7%). Gu et al. [20] measured the canal wall thickness at various levels in C-shaped root canals of mandibular first premolars and confirmed that the above findings were also applicable in mandibular first premolars with C-shaped canal. The minimum mesial wall thickness was frequently located at the lingual measurement site (67.3%). The irregular morphology of C-shaped canals would be more likely to result in strip perforation during root canal preparation [16, 32]. Nonlinear regression analysis in the present study revealed that the further the distance from the cementoenamel junction, the deeper the depth of the root radicular groove, and the smaller the angle, the thinner the mesial wall thickness. As a result, mechanical preparation of the C-shaped canal, particularly the lingual canal and the apical segment, must be executed carefully during treatment to prevent unexpected deviation and perforation.

High proportion of accessory canal (36.4%) and connecting canal (42.4%) increased root canal anatomy complexity. Most accessory canals (94.9%) were located in the middle and apical regions of the root, while the connecting canals were mainly located in the apical region (59.4%). The factors weaken the cleaning efficacy of the middle and apical segments of the root canal. The presence of a large number of accessory canals in the middle of the root significantly compromises the effectiveness of endodontic surgery, since accessory canals cannot be removed by 3 mm root cutting. Therefore, chemical irrigation and medication of the root canal system would be particularly important in the treatment to the special root canal anatomy.

The present data showed that the number of mandibular first premolar apical foramina was predominantly 1–2. With the variation in the numbers of apical foramina, there is a significant difference in the major diameter and minor diameter. To improve the cleaning of the apical region, the main file size should be wide enough to contact more canal walls [33]. In other words, the main file size should be larger than the maximum diameter to ensure that the root canal wall is fully cut. However, for oval root canals, prepping the root canal in a round shape might excessively remove the dentin and weaken the root [34]. Based on the measuring findings about the major and minor diameter, it was suggested that the main file numbers for mandibular first premolar teeth with C-shaped canal should be #35 for 1 apical foramen and #30 for 2 and 3 apical foramina. In the meantime, decreasing the taper of the files to avoid the perforation in the mesial wall of lingual canal also should be recommended.

## Conclusion

The findings of this study suggested that the incidence of C-shaped root canal system in mandibular first premolars was 16.9% in the Chinese population. The anatomy was extremely complex and variable, especially in the middle and the apical regions of the root canal. The thin mesial wall on the lingual side should be protected cautiously during instrumentation.

## Data Availability

All data generated or analyzed in this study are included in this published article.
